# A quality improvement comparative audit on theimpact of competency based basic intensive care medicine (ICM) training on the confidence levels of medical senior house officers registrars dealing with intensive care procedures in acutely ill patients

**DOI:** 10.1186/2197-425X-3-S1-A858

**Published:** 2015-10-01

**Authors:** V Gulia, A Goel, S Kumari, R Kumari, N Arora

**Affiliations:** Good Hope Hospital, Anaesthesia, Birmingham United Kingdom; North West Deanery, Manchester, United Kingdom; Good Hope Hospital, Birmingham, United Kingdom; City Hospital, Birmingham, United Kingdom

## Introduction

Medical training in the UK has now changed with the development of Foundation Programmes (which includes the pre-registration House Officer year plus one additional year) providing two years of experience in a range of disciplines that may include acute medicine and 3 months Basic training in ICM [1]. The ICM training programme, being competency based, can inter link well with these developments [2]. Intensive care has been described as a horizontal specialty cutting across the traditional vertical specialties of medicine, surgery, paediatrics etc [3]. Several studies have demonstrated the need for all medical postgraduates to undergo Intensive care Medicine training [4].

## Objectives

The aims of this audit were to reinforce the benefits and practicalities of basic level ICM training from a Medical trainee's perspective and summarize the key competencies for the assessment of the technical skills required to deal with acutely unwell patients in ward needing intensive Care.

## Methods

A questionnaire was sent out to all the Registrars and SHOs working in Medical Specialities (Cardiology , Renal Medicine , Haematology, Neurology, Acute Medicine , Respiratory Medicine etc) in West Midlands and Manchester region including ST and Staff grade level. 147 Registrars and SHOs in total responded to the questionnaire, 68 in Manchester and 79 in West Midlands. The questionnaire sought info about the prior involvement of the Registrars in formal 3 months Competency based ICM training at Basic Speciality level and the level of confidence in doing ICU procedures like Central line, Arterial line and managing invasive Ventilation, tracheostomy care and non invasive Ventilation and use of Vasopressors and Inotropes etc.

## Results

The registrars who had done ICM preliminary training for 3 months demonstrated a significant difference in being confident in inserting Central lines with Ultrasound guidance (p = 0.046) and non invasive Ventilation management (p = 0.009) than the group who did not do a formal ITU training . There was no significant difference in Confidence levels between the Medical trainees of various specialities .There was a significant difference in ease of using Inotropes and Vasopressors in the ICM trained group as compared to the non trained group with P = 0.008.

## Conclusions

It should be mandatory to do 3 months basic ICM competency before beginning Medicine Registrar position^5^. The confidence of dealing with ward patients is better in registrars who have completed competency based ICM basic training and helps in reducing burden on Intensive care registrars.
